# Lutein and Zeaxanthin Distribution in the Healthy Macula and Its Association with Various Demographic Factors Examined in Pseudophakic Eyes

**DOI:** 10.3390/antiox10121857

**Published:** 2021-11-23

**Authors:** Akira Obana, Yuko Gohto, Ryo Asaoka, Werner Gellermann, Paul S. Bernstein

**Affiliations:** 1 Department of Ophthalmology, Seirei Hamamatsu General Hospital, Hamamatsu City 430-8558, Shizuoka, Japan; yukogo@sis.seirei.or.jp (Y.G.); ryoasa0120@googlemail.com (R.A.); 2 Department of Medical Spectroscopy, Institute for Medical Photonics Research, Preeminent Medical Photonics Education & Research Center, Hamamatsu University School of Medicine, Hamamatsu City 431-3125, Shizuoka, Japan; 3 Longevity Link Corporation, Salt Lake City, UT 84108, USA; wbgellermann@gmail.com; 4 Department of Ophthalmology and Visual Sciences, Moran Eye Center, University of Utah School of Medicine, Salt Lake City, UT 84132, USA; paul.bernstein@hsc.utah.edu

**Keywords:** macular pigment, fundus autofluorescence spectroscopy, pseudophakia, age, smoking, body mass index, skin carotenoid levels

## Abstract

The macular pigment consisting of lutein (L) and zeaxanthin (Z) protects photoreceptors via its antioxidative and barrier activities. This study aimed to determine L and Z distribution in the healthy macula and their association with various demographic factors. Macular pigment optical density (MPOD) was measured using fundus autofluorescence spectroscopy in 352 pseudophakic eyes with no fundus diseases. Pseudophakia was chosen to avoid the influence of cataract in the measurement of fundus autofluorescence. The mean patient age was 72.3 ± 8.6 years. MPOD was analyzed separately in three zones, i.e., A: a central area within a radius of 0.5°, mainly containing Z; B: a ring area with radii from 0.5° to 1.3°, containing Z and L; C: a ring area with radii from 1.3° to 9°, containing L. Multivariate analyses were performed with MPOD as the dependent variable and sex, supplement intake, smoking habits, glaucoma, diabetes, age, body mass index (BMI), skin carotenoid levels, retinal thickness, retinal volume, axial length as the independent variables. The mean total MPOD volume within 9° eccentricity was 20,121 ± 6293. Age was positively associated with MPOD in all zones. Supplement and BMI were positively and negatively associated with MPOD in zones B and C. Smoking was negatively associated with MPOD in zone A. This study revealed the standard MP values of aged Japanese, which resulted to be higher than the previously reported values in other races. Age was found to have a positive association with MP values. L in the outer foveola was affected by BMI and supplements, but Z in the foveola was not. The amount of Z in the Müller cell cone may not be changed easily by factors such as hunger and satiety in the context of preservation of homeostasis in the human body, but tobacco had a negative effect on Z.

## 1. Introduction

The macular pigment (MP) consists of lutein (L) [(3R, 3′R, 6′R)-lutein] and two zeaxanthin (Z) stereoisomers, (3R, 3′R)-zeaxanthin and (3R, 3′S; *meso*)-zeaxanthin (*meso*-zeaxanthin) [1,2]. The MP absorbs short-wavelength visible light and filters blue light. The MP has an antioxidative effect, as it quenches oxygen radicals caused by blue light irradiation in the photoreceptors and retinal pigment epithelium (RPE) [3,4,5]. L also has an anti-inflammatory effect [6]. Oxidative damage and chronic inflammation are important factors causing age-related macular degeneration (AMD) [7,8,9,10,11,12], a major cause of legal blindness in the elderly. Therefore, the MP is an important physiological protection against AMD. Previous studies [13,14,15] showed low MPOD in AMD patients compared to healthy subjects, and we suggested low MPOD might be a risk factor for AMD development [16].

A histological study of the primate retina [17] showed that MP mainly localizes in the outer plexiform layer at the fovea (Henle layer) and partly in the inner plexiform layer. MP also extends vertically through all layers of the retina in the foveal center. The particular distribution of MP in the healthy Japanese retina has been investigated [18]. The distribution pattern of MP could be divided roughly into two regions: MP in the fovea within 0.5° eccentricity, and MP surrounding this area. The area within 0.5° lacks the inner layers of the retina, i.e., those from the inner nuclear layer to the nerve fiber one, and we consider that MP within this area exists in the Müller cell cone according to the hypothesis of Gass [19]. Investigations on retinal diseases, including macular telangiectasia type 2 [20,21,22], lamellar macular hole [23], and macular hole [24,25], support this assumption. A recent study using Raman microscopy revealed a more detailed distribution of MP in human histological specimens [26]. According to this study, Z accumulates in the anatomical foveola at a high concentration, which drops sharply at the periphery near the macula. Conversely, L distributes across the macula more evenly and at lower concentrations. The Z/L ratio can be greater than 9:1 in the foveal center, is 4:1 in a spot around 200 μm from it, and is about 1:4200 μm farther. Considering this distribution, the MP in the Müller cell cone within 0.5° eccentricity consists of Z (including both isomers), and the MP surrounding the area consists of Z and L. The absolute amount of Z and the Z/L ratio decrease with eccentricity.

The MP and its distribution can be measured in the eye using fundus autofluorescence (AF) spectroscopy. Measurements are obtained from the extent of attenuation of RPE lipofuscin AF by the MP [27]. Most commonly, dual-wavelength AF spectroscopy is performed using the Spectralis imaging platform (Heidelberg Engineering, Heidelberg, Germany). The accuracy of this device has been validated by comparison with heterochromatic flicker photometry (HFP), a widely utilized psychophysical technique [28,29,30,31,32,33]. However, fundus AF spectroscopy has a shortcoming: cataractous lenses can attenuate MPOD levels by absorbing and scattering the excitation and emission light, especially the blue wavelengths. Some correction methods for opacity-induced MPOD level attenuations have been presented [34,35,36,37], but such models have limitations. Therefore, limiting the measurement of MPOD to eyes with an intraocular lens (IOL) can be useful in clinical studies, especially in aged subjects.

In this study, MPOD levels in the healthy macula were measured using dual-wavelength AF spectroscopy in eyes with IOLs to avoid the influence of cataracts. As far as we are aware of, this study presents the largest collection of MPOD data of pseudophakic eyes, and the present data will be valuable as standard MPOD levels in Japanese for future studies on the MP. Many clinical and demographic factors have been associated with MPOD levels [38], but there are some inconsistencies between studies. Such associations are re-evaluated in this study, with special attention to regions that are rich in either Z or L.

## 2. Subjects and Methods

The institutional review board of Seirei Hamamatsu General Hospital approved this observational study (IRB No. 2199, 2251). The study followed the tenets of the Declaration of Helsinki. All patients provided written informed consent for measuring their MPOD and skin carotenoid (SC) levels prior to cataract surgery.

### 2.1. Subjects

**Figure 1:**
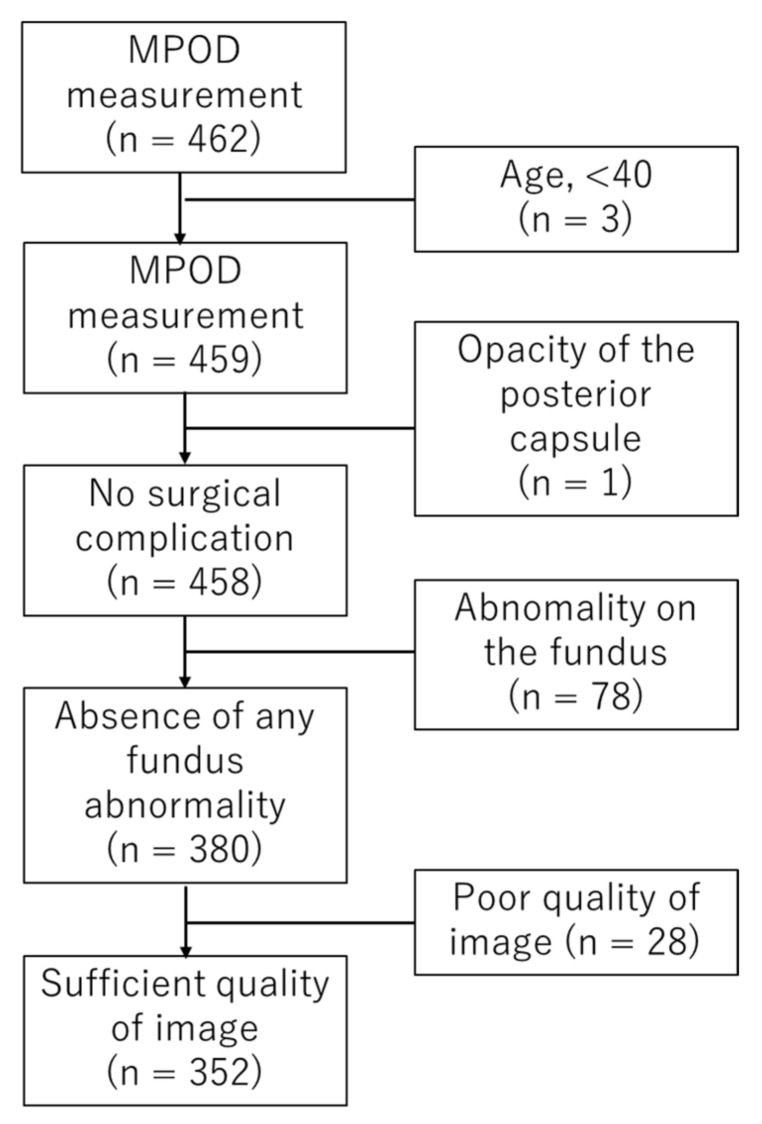
Flow diagram of subject selection. In total, 355 eyes were selected from 462 eyes. N indicates the number of eyes.

After measuring MPOD in 462 eyes between 3 and 5 days after cataract surgery, patients aged less than 40 years were excluded. Then, 352 eyes of 242 patients were enrolled in this study. Figure 1 shows the subject selection process. Patients were 41–91 years old, with a mean age of 72.3 ± 8.6 (standard deviation, SD) years. The median was 74 years. Men were 108 (155 eyes), and women were 134 (197 eyes). Some eyes were described in our previous manuscripts and were analyzed in different ways [18,31,36,37,39,40].

All subjects underwent cataract surgery at Seirei Hamamatsu General Hospital between October 2016 and March 2021. Cataract surgeries were performed using phaco instruments (Infinity or Centurion, Alcon Japan Ltd, Tokyo, Japan.) and an operating microscope (OPMI Visu210/S88, Carl Zeiss, Oberkochen, Germany) under sub-Tenon anesthesia. The operating technique was the same reported in the previous study [40]. IOLs were implanted in the capsular bag in all eyes. A yellow-tinted IOL was implanted in all eyes except for six eyes that received clear IOLs. Axial length was measured before the operation using an optical biometer (OA-2000, TOMEY, Nagoya, Japan). Visual acuity was measured by a decimal visual acuity test chart (K-3437, Inami, Tokyo, Japan). Intraocular pressure measurement, ophthalmological examinations, and measurements of MPOD and skin carotenoid (SC) levels were performed between 3 and 5 days after the operation. The absence of fundus diseases was confirmed after mydriasis with 2.5% phenylephrine hydrochloride and 1% tropicamide using indirect ophthalmoscopy, slit-lamp microscopy with a widefield noncontact lens, and optical coherence tomography (OCT). Spectralis-OCT (Heidelberg Engineering, Heidelberg, Germany) and Nidek RS-3000 OCT (Nidek, Aichi, Japan) were used for 341 and 11 eyes, respectively. Eyes with epiretinal membranes causing an abnormal contour of the normally smooth foveal depression were excluded. The software of both OCT machines calculates retinal thickness and volume based on an Early Treatment Diabetic Retinopathy Study (ETDRS) grid [41]. The mean retinal thickness within a radius of 0.5 mm (central retinal thickness, CRT) was obtained using the software of either OCT. The retinal volume within a radius of 0.5 mm from the central area (central retinal volume, CRV), the retinal volume in the ring from delimited by a radius of 0.5–3 mm (paracentral retinal volume, PRV), and the total retinal volume at a radius of 3 mm diameter from the central area (total retinal volume, TRV) were obtained using Spectralis-OCT software.

### 2.2. Measurement of Macular Pigment Optical Density

MPOD levels were measured using a prototype MPOD module installed on a Spectralis-OCT. This device uses 486 nm and 517 nm excitation wavelengths. The basic functionality and handling of this instrument have been described in more detail elsewhere [33,35,36]. The reference point was 9°, above which the plotted MPOD levels were no longer discernable from the noise [42]. Blue light absorption by the IOLs does not affect MPOD because the AF spectroscopy calculates the MPOD value from the comparison of AF intensities between the target with MP and the background without MP that are equally affected by yellow-tinted IOLs. Coordinating with the regions investigated in other studies, the average optical densities at 0.23°, 0.5°, 1°, and 2° eccentricities [MPOD(0.23), MPOD(0.5), MPOD(1), MPOD(2)] and the MP optical density volume (MPOV) within the boundary of 0.5°, 1°, and 2° eccentricities and 9° eccentricity [MPOV(0.5), MPOV(1), MPOV(2), MPOV(9)] were analyzed. Additionally, MPOD at 1.3° eccentricity [MPOD(1.3) and MPOV(1.3)] were analyzed to correlate the results with those of a recent histological investigation [26].

**Figure 2:**
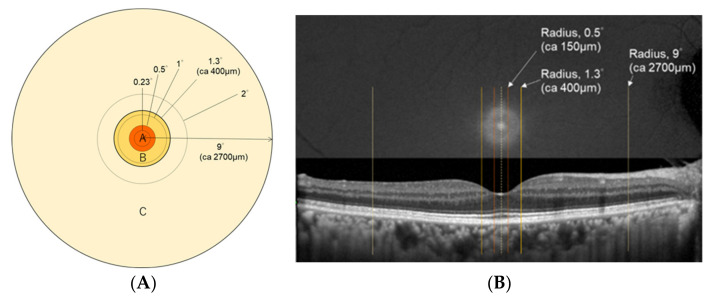
(**A**) Zone differentiation of MPOD measurement points. Zone A represents the area within a radius of 0.5°. Zone A contains mostly Z. Zone B represents a ring with radii of 0.5° and 1.3°. Zone B contains both Z and L. Zone C represents the area delimited by radii of 1.3° and 9°. Zone C contains mostly L. (**B**) Location of the three zones on macular pigment and OCT images. Images are from a 62-year-old-female, right eye. The central line indicates the center of the foveola. The following lines indicate the areas delimited by the mentioned radii, corresponding to each zone.

The area within 9° eccentricity was divided into three zones (Figure 2A). Zone A was the area within a radius of 0.5° that corresponded to the anatomical foveola, zone B was a ring delimited by radii of 0.5° and 1.3° that corresponded to the inner half of the anatomical fovea, and zone C was a ring delimited by radii of 1.3° and 9° that corresponded to the anatomical macula. According to Li et al. [26], zone A contains mostly Z, zone B contains both Z and L, and zone C contains mostly L. Therefore, MPOD(0.23), MPOD(0.5), and MPOV(0.5) contain mostly Z, MPOD(2) and MPOV(1.3–9) contain mostly L, while MPOD(1) and MPOD(1.3) contain a mixture of Z and L. Each location was projected on the MP and OCT images for identifying the exact sites (Figure 2B).

### 2.3. Evaluation of Skin Carotenoid (SC) Status

The measurement of serum L and Z concentration is a standard way to determine the systemic carotenoid status. However, the measurement of L and Z serum concentration is mildly invasive and time- and cost-consuming. Recent studies have shown that SC levels can be measured noninvasively and have a high correlation coefficient with serum carotenoid concentration [43,44,45,46,47,48,49,50,51]. Therefore, the SC level was used to determine the systemic carotenoid status instead of measuring L and Z serum concentration. SC levels were measured on the left middle finger by pressure-mediated reflection spectroscopy (RS) (Veggie Meter, Longevity Link Corporation, Salt Lake City, UT, USA). The basics of this device have been described elsewhere [52]. Calibration was performed before starting daily skin measurements, repeated every 3 h. The SC index was determined as the average of three consecutive measurements.

### 2.4. Statistical Analyses

Multivariate analyses were performed with MPOD and MPOV as dependent variables and categorial (sex, supplement intake, smoking habits, glaucoma, and diabetes) and continuous (age, BMI, SC levels, CRT, CRV, PRV, TRV, axial length) parameters as independent variables by a mixed linear regression model with patients as a random effect because one or two eyes of a patient were included. The fit of the regression equation was evaluated using the marginal R-squared (mR^2^) value, following a method proposed by Nakagawa and Holgerby [53]. In patients whose both eyes were included, SC measurement was performed twice, corresponding to MPOD measurement on both eyes. The mean SC values of two measurements were used for further analyses. All statistical analyses were performed using the statistical programming language R (ver. 3.6.1, The R Foundation for Statistical Computing, Vienna, Austria).

## 3. Results

### 3.1. Subject Characteristics

Table 1 and Table 2 show the patient and subject eye characteristics. In total, 132 patients received cataract surgery in only one eye, and 110 patients received it in both eyes. Eighteen patients reported taking L-containing supplements. Most L supplements provided in Japan contain only L, while a few products contain L and Z, but none of them contain other carotenoids. Detailed information about the exact dosages and formulations was not fully obtained.

### 3.2. Macular Pigment Optical Density and Macular Pigment Optical Volume

Table 3 shows the range and mean values of MPOD at five eccentricities and MPOV within five eccentricities.

### 3.3. Multivariate Analyses

Table 4 displays the results of multivariate analysis. Age had a significant positive association with MPOD and MPOV in all zones. BMI did not have a significant association with MPOD and MPOV in zone A but had a significantly negative association with MPOD and MPOV in zones B and C. Supplements had a significantly positive association with MPOD and MPOV in zone C and MPOD(1.3). Smoking had a significantly negative association with MPOD and MPOV in zone A. Other factors, including sex, SC levels, glaucoma, diabetes, axial length, CDT, CRV, PRV, and TRV, had no significant associations with MPOD and MPOV in all zones.

**Figure 3:**
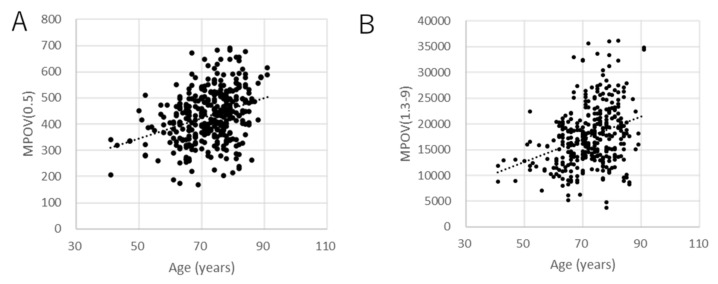
Scatter plots of MPOV against age. (**A**) MPOV(0.5) (coefficient = 3.47, *p* < 0.001, linear mixed model), (**B**) MPOV(1.3–9) (coefficient = 209.3, *p* < 0.001, mixed linear model).

**Figure 4:**
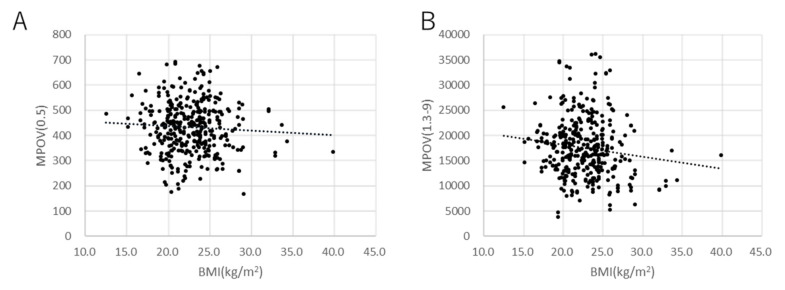
Scatter plots of MPOV against BMI. (**A**) MPOV(0.5) (coefficient = −2.17, *p* = 0.30). (**B**) MPOV(1.3–9) (coefficient = −311.8.0, *p* = 0.010, mixed linear model).

**Figure 5:**
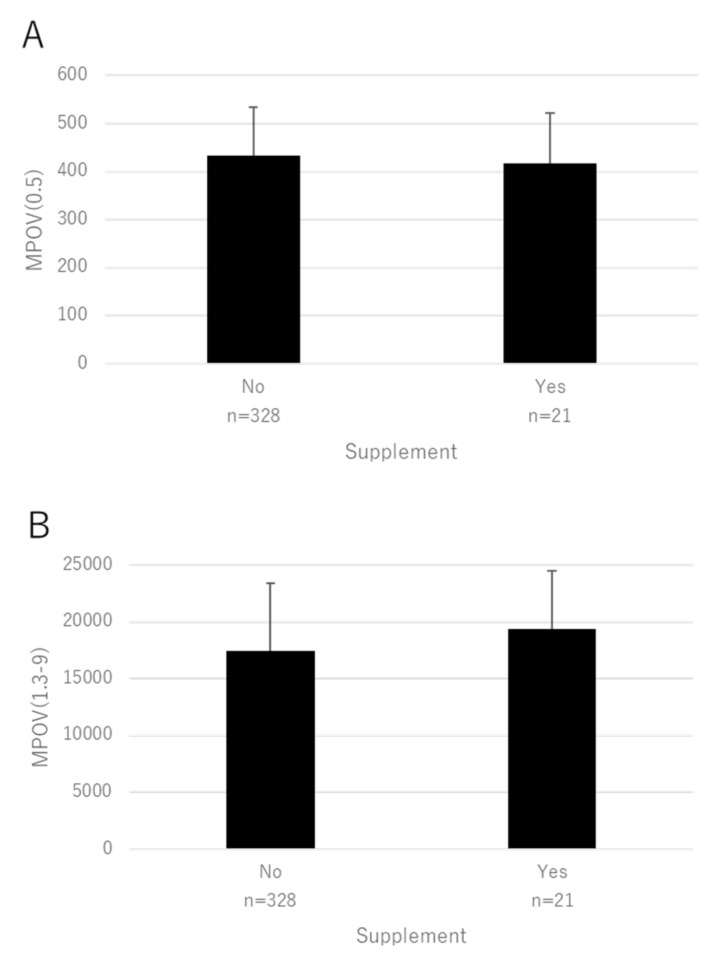
Mean MPOV in patients with respect to supplement intake. (**A**) MPOV(0.5) (*p* = 0.33). (**B**) MPOV(1.3–9) (*p* = 0.57, mixed linear model). Bar represents standard deviation.

**Figure 6:**
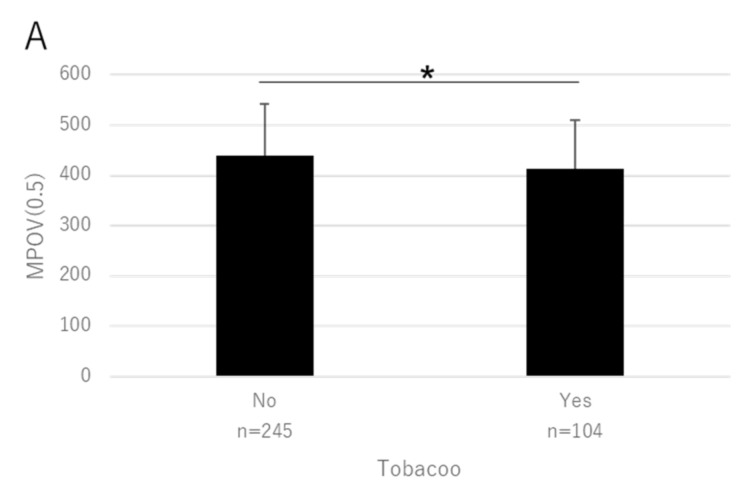

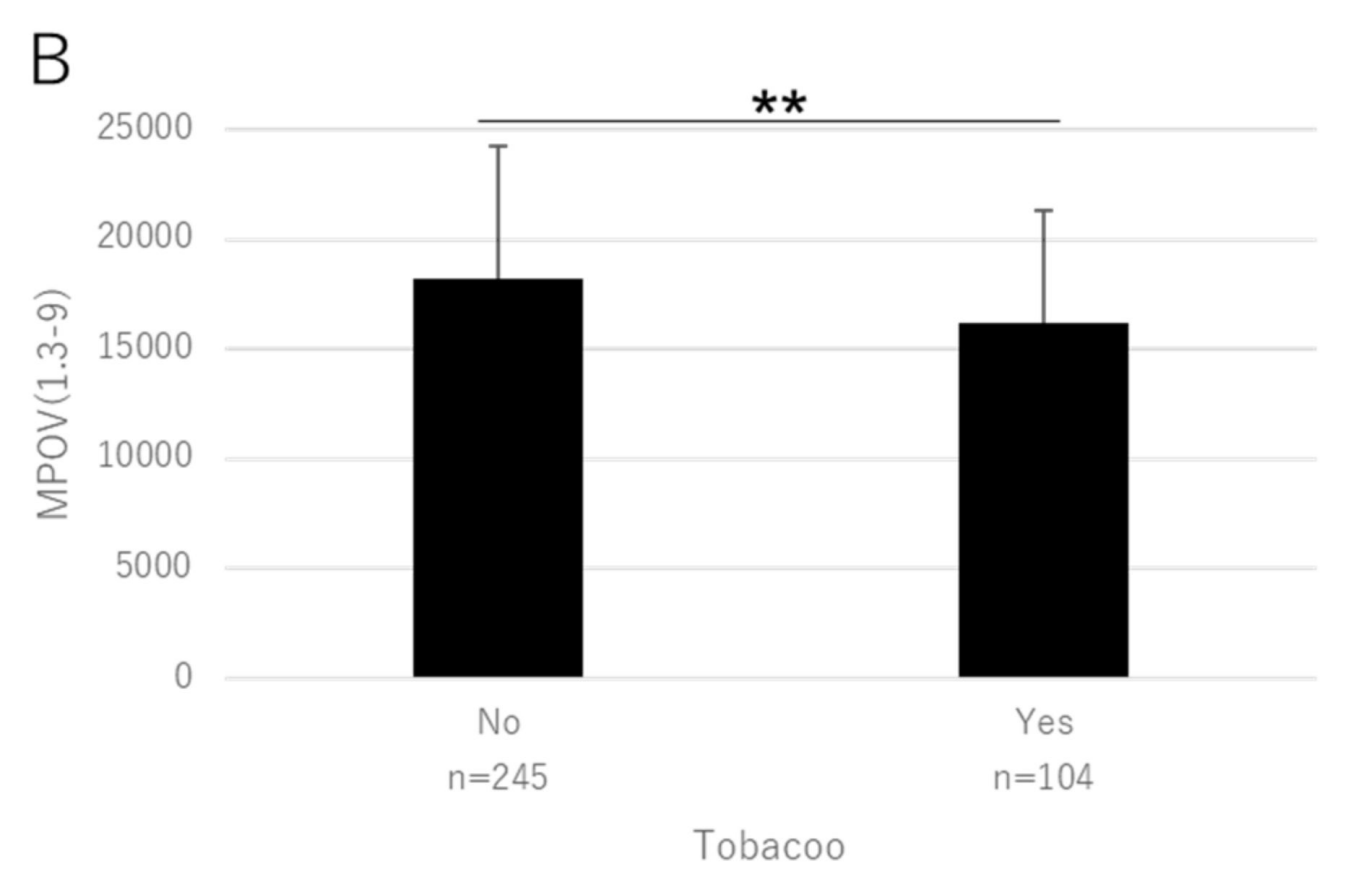
Mean MPOV in patients in relation to their smoking history. (**A**) MPOV(0.5) (* *p* = 0.0040). (**B**) MPOV(1.3–9) (** *p* = 0.0010, mixed linear model). Bar represents standard deviation.

Scatter plots of factors that were significant in the multivariate analyses (age, BMI, supplement, and smoking) are shown in Figure 3, Figure 4, Figure 5 and Figure 6 for MPOV(0.5) and MPOV(1.3–9).

## 4. Discussion

The present results showed MPOD values not influenced by cataracts in aged subjects. These values could represent a standard for future investigation on MP. MPOD(0.23), MPOD(0.5), MPOD(1), MPOD(2), and MPOV(9) were consistent with our previous studies [18,31,36,37,39,40], and this consistency was expected, since the current study was based in part on the same database, although the number of subjects in the present study was larger. The MPOD levels were higher in areas closer to the foveal center. In the case of 0.23° eccentricity, the mean MPOD was 0.78, which means that MP (mostly Z contained in the Müller cell cone) blocked the blue light by 83.4%. Previous studies have reported mean MPOD levels within 2° eccentricity measured by the same method mainly in Caucasians [29,32,54], and those values were lower than the present values. However, a direct comparison was impossible due to the different reference points. The average of MPOV(9) was 20,121. Previous studies reported that the average MPOV within 7° eccentricity was 5094 (*n* = 393) and 4729 (*n* = 97) in the Irish population and 8307 (*n* = 215) in the Mexican population [42,55]. In order to compare these results directly, we calculated MPOV using our present data with a reference point of 7°. The average of MPOV(7) was 12,135 ± 3718, which was higher than that of Irish and Mexicans. A reason for the present high MP levels was that all the present data were from pseudophakic eyes. In our previous study [37], the mean error for MPOV in eyes with cataract was 20.5% without any correction. When the values by Green-Gomez et al. are corrected with this factor, MPOV(7) is 10,010, which is still lower than our present value. Racial differences could account for this discrepancy. Studies have shown that non-White populations have higher MPOD levels than White populations [38,56,57]. 

Age had a significant positive association with MP levels in all regions when considering the limited age range of patients in this study. MPOD in subjects younger than 40 years is still unknown. Age is a controversial factor when determining MP levels. Some studies have shown an age-related decline of MPOD levels [15,16,58,59,60], and others have shown no change [61,62,63] or an age-related increase [64]. Our previous study using resonance Raman spectroscopy to measure MPOD [65] indicated an age-related decline in MPOD in 144 pseudophakic eyes in patients in an age range of 55–88 years (average: 73.7 ± 7.3 years). Raman spectroscopy evaluates total MPOD values within a 1 mm-diameter laser spot aimed at the fovea, which is equivalent to MPOV(1.3) in the present study. The partial regression coefficient of multivariate analysis on MPOV(1.3) was 23 (*p* < 0.001; data not shown). Reasons for the discrepancy between the two studies are unclear, but the methodology used would be a major reason. According to the studies with OCT, CRT increases with age, but the thickness of the outer fovea decreases with age because of the decreased thickness of the inner retina [66]. This age-related decrease in the inner retina may influence AF intensity at the reference point (9° eccentricity), since scattering of blue excitation light is reduced. Dual-wavelength AF spectroscopy calculates log[(AF_G_-background offset)/(AF_B_-background offset)] (AF_G_ is AF intensity by green light excitation, AF_B_ is AF intensity by blue light excitation) at the target point and reference point, and MPOD is obtained by the subtraction of these values at the two points. If AF_B_ becomes large due to less scattering of the excitation light, log[(AF_G_-background offset)/(AF_B_-background offset)] at the reference point decreases, and MPOD increases. Therefore, it is possible that the evaluation of MPOD supplied higher values than the actual values in aged subjects. This hypothesis must be verified further. Another reason is the limitation of the cross-sectional design of the present and previous studies; therefore, a longitudinal study is needed. 

BMI had a negative association with MPOD and MPOV in zones B and C. Lipophilic L is stored in the adipose tissue, and a tissue interaction or competition for L between adipose tissue and the retina has been postulated [67]. An inverse association between BMI and MP has been reported by others [61,67,68,69].

L- and Z-containing supplements are well known to increase MPOD levels. This effect was confirmed for L supplements provided in Japan [70,71,72]. After taking the L supplement, serum L concentration and SC levels increase. SC levels highly correlate with serum carotenoid levels [43,44,45,46,47,48,49,50,51]. However, the increase in MPOD is slower than the increase in SC levels [72]. Previous studies showed a statistically positive association between serum L and Z concentrations and MPOD [61,71,72,73,74,75]. Still, the correlation was weak, with low synchronicity [76] because of the accumulation tendency of MP. Moreover, the serum concentrations and MPOD responses to L-containing supplements varied among individuals [70,77]. This weak correlation with serum concentration may be the reason why the SC levels showed no significant association with MPOD and MPOV in this study. 

This study showed a unique association between MPOD and BMI or supplement intake. MPOD and MPOV in zones B and C were associated with BMI and supplement intake; however, MPOD and MPOV in zone A were not associated with them. Another study in Japanese patients [71] reported a similar trend. It showed MPOD at 2° eccentricity was significantly associated with serum L + Z concentrations, but MPOD at 0.25° and 0.5° eccentricity was not associated with them. These results suggested that L in the parafoveal region depends on the serum concentration, but Z in the Müller cell cone in the central lesion hardly depends on the serum concentration. The Müller cell cone has an essential role in blocking blue light directed to cone photoreceptors. Therefore, the amount of Z in the Müller cell cone may not be changed easily by factors such as hunger and satiety in the context of preservation of homeostasis in the human body; however, this speculation needs further investigation.

Smoking history had a negative association only in zone A. Previous studies also showed a negative effect of smoking on MPOD [58,78], but some studies failed to show a significant association [65,79,80,81]. The influence of tobacco might on Z and L be different, as suggested by the present results. 

This study has several limitations. L and Z intake from dietary food was not evaluated, and comprehensive supplement histories were not obtained. The evaluation of serum concentrations of L and Z was replaced by SC measurement. The retinal thickness of the three zones could not be determined because the ready-made program of OCT did not match zone classification.

## 5. Conclusions

This study revealed the standard MP values of aged Japanese based on a large sample of pseudophakic eyes. Age was found to have a positive association with MP values, but this issue needs further verification. BMI had a negative association with MP in the area beyond 0.5° eccentricity, and L supplements were positively associated with MP in the same area. However, BMI and supplements had no significant association with MP in the foveola (within 0.5° eccentricity). These results suggest that L in the outer foveola is affected by BMI and supplements, but Z in the foveola is not. Since Z in the foveola has to block blue light directed to cone photoreceptors, human eyes may keep consistent MP levels in the Müller cell cone. Tobacco had a negative association with Z, but the association with L was not confirmed statistically.

## 6. Patents

Bernstein PS, Gellermann W, McClane RW (1999). Method and system for the measurement of macular carotenoid levels. U.S. Patent # 5,873,831.

Gellermann W, McClane RW, Katz NB, Bernstein PS (2009). Method and apparatus for the noninvasive measurement of carotenoids and related chemical substances in biological tissue. Japan Patent # 4336673.

Ermakov IV, Gellermann W. Noninvasive Measurement of Carotenoids in Biological Tissue. U.S. Patent # 8,260,402 granted 2012, Japanese Patent JP 5574246B2 granted 2014.

## Figures and Tables

**Table 1:** Patient characteristics.

Characteristics	Number of Patients
Enrolled eyes	One eye, 132, Both eyes, 110
Sex	Men, 108, women, 134
Age (years)	Range, 41–91, mean, 72.3 ± 8.6 (standard deviation), median, 74
Body mass index (kg/m^2^)	Range, 12.5–39.9, mean, 22.7 ± 3.3 (standard deviation)
Tobacco	No, 170, Yes, 70 (Past 52/Current18), unknown, 2
Lutein supplement	No, 222, Yes, 18, unknown, 2
Diabetes	No, 205, Yes, 37, unknown, 3

**Table 2:** Characteristics of the subject eyes.

Characteristics	
Axial length (mm)	Range, 20.8–28.9, mean, 23.9 ± 1.4 (standard deviation)
Central retinal thickness, CRT (µm)	Range, 214–381, mean, 267.7 ± 22.7
Central retinal volume, CRV (mm^3^)	Range, 0.17–0.30, mean, 0.20 ± 0.02
Paracentral retinal volume, PRV (mm^3^)	Range, 6.82–9.53, mean, 8.26 ± 0.44
Total retinal volume, TRV (mm^3^)	Range, 7.02–9.79, mean, 8.47 ± 0.45
Glaucoma	No, 328, Yes, 25
BCVA at the MPOD measurement	1.2 (logMAR −0.08), 283 1.0 (logMAR 0.00), 39 0.9 (logMAR 0.05), 10 0.8 (logMAR 0.10), 13 0.7 (logMAR 0.15), 4 0.6 (logMAR 0.22), 2 0.5 (logMAR 0.30), 1

BCVA: best corrected visual acuity; MPOD: macular pigment optical density; MAR: minimum angle resolution.

**Table 3:** Range and mean ± standard deviation of MPOD at five eccentricities and MPOV within five eccentricities.

Ecentricity	Local MPOD	MPOV
0.23°	0.24–1.24 0.78 ± 0.18	
0.5°	0.25–1.19 0.73 ± 0.18	169–692 432 ±101
1°	0.22–1.17 0.69 ± 0.16	519–2358 1490 ± 331
1.33°	0.15–1.05 0.55 ± 0.15	832–5179 2542 ± 600
2°	0.08–0.74 0.35 ± 0.11	1289–8244 44,462 ± 1125
9°	-	5100–40,224 20,121 ± 6293

**Table 4:** Multiple regression analysis for MPOD and MPOV classified into three zones.

		Zone A			Zone B		Zone C	
		MPOD(0.23)	MPOD(0.5)	MPOV(0.5)	MPOD(1)	MPOV(1.3)	MPOD(2)	MPOV(1.3–9)
mR^2^		0.24	0.25	0.26	0.24	0.27	0.24	0.20
Age (years)	B	**6.5 × 10^−3^**	**6.8 × 10^−3^**	**4.0**	**7.9 × 10^−3^**	**7.3 × 10^−3^**	**4.6 × 10^−3^**	**2.4 × 10^2^**
	β	**5.7 × 10^−3^**	**6.0 × 10^−2^**	**3.5 × 10^1^**	**6.9 × 10^−2^**	**6.3 × 10^−2^**	**4.0 × 10^−2^**	**2.1 × 10^3^**
	p	**<0.001**	**<0.001**	**<0.001**	**<0.001**	**<0.001**	**<0.001**	**<0.001**
Sex (0 woman, 1 man)	B	2.1 × 10^−2^	1.0 × 10^−2^	1.5 × 10^1^	7.9 × 10^−3^	1.4 × 10^−2^	4.1 × 10^−3^	5.5 × 10^1^
	p	0.444	0.707	0.487	0.747	0.548	0.814	0.956
BMI (kg/m^2^)	B	−5.2 × 10^−3^	−4.8 × 10^−3^	−2.9	**−7.6 × 10^−3^**	**−6.7 × 10^−3^**	**−5.3 × 10^−3^**	**−3.3 × 10^2^**
	β	−1.6 × 10^−2^	−1.5 × 10^−2^	−9.1	**−2.3 × 10^−2^**	**−2.1 × 10^−2^**	**−1.6 × 10^−2^**	**−1.0 × 10^3^**
	p	0.133	0.159	0.128	**0.017**	**0.023**	**0.020**	**0.010**
Supplement (0 no, I yes)	B	−8.1 × 10^−4^	−4.3 × 10^−2^	−5.0	5.3·10^−2^	**6.3 × 10^−2^**	**6.0 × 10^−2^**	**3.8 × 10^3^**
	p	0.984	0.277	0.824	0.112	**0.040**	**0.010**	**0.004**
Smoking (0 no, I yes)	B	**−5.5 × 10^−2^**	**−6.4 × 10^−2^**	**−3.6 × 10^1^**	−4.4 × 10^−2^	−3.6 × 10^−2^	−3.0 × 10^−2^	−1.3 × 10^3^
	p	**0.045**	**0.020**	**0.020**	0.079	0.128	0.100	0.207
SC levels	B	7.4 × 10^−5^	1.4 × 10^−4^	4.9 × 10^−2^	1.1 × 10^−4^	1.0 × 10^−4^	7.6 × 10^−5^	5.1
	β	1.1 × 10^−2^	2.0 × 10^−2^	7.0	1.5 × 10^−2^	1.5 × 10^−2^	1.1 × 10^−2^	7.3 × 10^2^
	p	0.331	0.072	0.252	0.115	0.098	0.112	0.061
Glaucoma (0 no, I yes)	B	−7.7 × 10^−2^	−6.4 × 10^−2^	−4.4 × 10^1^	−3.1 × 10^−2^	−2.1 × 10^−2^	−5.2 × 10^−3^	1.5 × 10^2^
	p	0.064	0.120	0.059	0.415	0.551	0.848	0.919
Diabetes (0 no, I yes)	B	−1.9 × 10^−2^	−1.4 × 10^−2^	−9.1	−2.0 × 10^−3^	6.4 × 10^−3^	1.3 × 10^−2^	2.2 × 10^2^
	p	0.513	0.636	0.585	0.941	0.803	0.517	0.844
Axial length (mm)	B	−6.7 × 10^−3^	6.5 × 10^−3^	−6.9 × 10^−2^	5.1 × 10^−3^	−5.2 × 10^−3^	−3.3 × 10^−3^	−1.2 × 10^2^
	β	−9.5 × 10^−3^	9.2 × 10^−3^	−9.8 × 10^−2^	7.2 × 10^−3^	−7.3 × 10^−3^	−4.6 × 10^−3^	−1.7 × 10^2^
	p	0.480	0.487	0.990	0.530	0.491	0.563	0.698
CRT (µm)	B	3.4 × 10^−3^	2.3 × 10^−3^	1.70	−1.0 × 10^−4^	−1.5 × 10^−3^	−7.2 × 10^−4^	−3.2 × 10^1^
	β	7.7 × 10^−2^	5.1 × 10^−2^	3.8 × 10^1^	−2.3 × 10^−3^	−3.4 × 10^−2^	−1.6 × 10^−2^	−7.3 × 10^2^
	p	0.080	0.195	0.110	0.933	0.161	0.353	0.434
CRV (µm^3^)	B	−4.4 × 10^−1^	2.9·10^−1^	−3.3 × 10^1^	6.5 × 10^−1^	1.5	6.9 × 10^−1^	3.2 × 10^4^
	β	−8.0 × 10^−3^	5.1·10^−3^	−5.9 × 10^−1^	1.2 × 10^−2^	2.5 10^−2^	1.2 × 10^−2^	5.7 × 10^2^
	p	0.846	0.888	0.979	0.634	0.226	0.427	0.489
PRV (µm^3^)	B	−2.1 × 10^−1^	−3.5 × 10^−1^	−1.8 × 10^2^	2.8 × 10^−1^	5.2 × 10^−1^	2.9 × 10^−1^	1.6 × 10^4^
	β	−9.2 × 10^−2^	−1.5 × 10^−1^	−8.1 × 10^2^	1.2 × 10^−1^	2.3 × 10^−1^	1.3 × 10^−1^	7.1 × 10^3^
	p	0.787	0.610	0.662	0.554	0.197	0.327	0.304
TRV (µm^3^)	B	9.5 × 10^−2^	2.4 × 10^−1^	1.2 × 10^2^	−3.3 × 10^−1^	−5.5 × 10^−1^	−3.2 × 10^−1^	−1.6 × 10^4^
	β	4.3 × 10^−2^	1.1 × 10^−1^	5.3 × 10^1^	−1.5 × 10^−1^	−2.5 × 10^−1^	−1.4 × 10^−1^	−7.2 × 10^3^
	p	0.902	0.730	0.779	0.484	0.178	0.280	0.308

MPOD: macular pigment optical density; MPOV: macular pigment optical volume; BMI: body mass index; SC levels: skin carotenoid levels; CRT: central retinal thickness; CRV: central retinal volume; PRV: perifoveal retinal volume; TRV: total retinal volume; B: partial regression coefficient; β: standardized partial regression coefficient.

## Data Availability

Data available on request due to restrictions eg privacy or ethical. The data presented in this study are available on request from the corresponding author. The data are not publicly available because they include patients’ personal information.
